# A Novel FEM-Based Numerical Solver for Interactive Catheter Simulation in Virtual Catheterization

**DOI:** 10.1155/2011/815246

**Published:** 2011-12-08

**Authors:** Shun Li, Jing Qin, Jixiang Guo, Yim-Pan Chui, Pheng-Ann Heng

**Affiliations:** ^1^The Department of Computer Science and Engineering, The Chinese University of Hong Kong, Hong Kong; ^2^Shenzhen Institutes of Advanced Technology, Chinese Academy of Sciences, Hong Kong

## Abstract

Virtual reality-based simulators are very helpful for trainees to acquire the skills of manipulating catheters and guidewires during the vascular interventional surgeries. In the development of such a simulator, however, it is a great challenge to realistically model and simulate deformable catheters and guidewires in an interactive manner. We propose a novel method to simulate the motion of catheters or guidewires and their interactions with patients' vascular system. Our method is based on the principle of minimal total potential energy. We formulate the total potential energy in the vascular interventional circumstance by summing up the elastic energy deriving from the bending of the catheters or guidewires, the potential energy due to the deformation of vessel walls, and the work by the external forces. We propose a novel FEM-based approach to simulate the deformation of catheters and guidewires. The motion of catheters or the guidewires and their responses to every input from the interventionalist can be calculated globally. Experiments have been conducted to validate the feasibility of the proposed method, and the results demonstrate that our method can realistically simulate the complex behaviors of catheters and guidewires in an interactive manner.

## 1. Introduction

Vascular interventional radiology (VIR) [1] is a minimally invasive surgery (MIS) procedure. It has been widely used to cure vascular diseases, such as stroke, angiostenosis, and aneurysm. This therapy is performed by using two main kinds of instruments, catheters, and guidewires (for brevity, we use “catheters” to represent “catheter, and guidewires” hereafter) both of which are very flexible cylindrical instruments. In the procedures, they are inserted in the patient's vascular system and driven by the interventionalists to the desired point. This task is complicated because only 2D X-ray images are available, and the catheters have to be handled by the tail. It becomes a challenge to train the novices to let them acquire the skills for safe and efficient procedures [2].

There are some traditional methods for the training of catheterization skills, where the trainees practice on animals, alternative anatomic phantoms, or even actual patients. Due to distinct anatomical differences between animals' and human beings' vascular network, animals are not good substitutes of human beings for training. On the other hand, it is very difficult and expensive to produce a phantom with complex blood vessels the same as real patients. Operating on real patients directly cannot be acceptable as well, as it is very dangerous to both patients and trainees. In contrast to these traditional training methods, virtual reality- (VR-) based simulation systems provide a promising way for catheterization training with high flexibility, high realism, and low cost while without risks to patients and trainees, and there have been some research works on developing such systems in the last few years [3–6]. In order to provide a virtual training environment, a simulator would be developed to simulate the behavior of the catheters navigating inside the patient's vascular system. Therefore, the position of the catheter inside the blood vessel and its changes by the operations from the interventionalists, such as pulling or pushing, ought to be figured out by a numerical algorithm.

Several methods have been proposed to simulate the behaviors of catheters in catheterization procedures. Dawson et al. [7] firstly employed a set of rigid links connected by joints to simulate catheters where the catheter was moved by three forces, such as contact force, injection force, and forces exerted by users. However, this model cannot realistically simulate the complex behaviors of catheters in catheterization. Later, Wang et al. [8] developed a mass-spring model for catheter simulation dynamically, but it is not consistent with physics laws of elastic thin objects. Cotin et al. [9–11] model the catheter with a set of linked deformable beams. They proposed an incremental finite element method (FEM) built on the strain-stress model of the beams for catheter simulation. Because of the local and incremental characteristics of their approach, the local errors generated when calculating the displacements of the beams can also be translated incrementally, and it is rather difficult to restrict the total error to an acceptable level. An relatively accurate model was proposed in the work of Alderliesten et al. [12, 13], which resorts to the principle of energy minimization to figure out the equilibrium of the catheters, and a semianalytic method is developed to solve this model. However, its computational cost is too expensive to be acceptable for an interactive simulator. Recently, Tang et al. [14] developed a simulating approach based on the work of Bergou et al. [15], where the virtual catheter was driven by elastic forces acted on each node of a discrete catheter. However, the stability and accuracy of this simulator is restricted by the time step used in the numerical solver.

Inspired by the methods proposed by Alderliesten et al. [12, 13], in this paper, we propose a novel method to simulate the motion of catheters and their interactions with patients' vascular system based on the principle of minimal total potential energy. We formulate the total potential energy in the vascular interventional circumstance by summing up the elastic energy deriving from the bending of the catheters, the potential energy due to the deformation of the vessel wall, and the work by the external forces. In order to overcome the shortcoming of expensive computational costs of the method by Alderliesten et al., we proposed a novel FEM-based approach to figure out the deformation of catheters while interacting with the blood vessel wall, which transforms the problem of minimizing the energies to solving a linear system. Thus, the motion of the catheter and its responses to every input from the interventionalist can be calculated globally. Our method provides a good trade-off between the accuracy and efficiency; that is, our method can achieve relatively accurate simulation while maintaining interactive performance. Comparing with other interactive simulating methods, since our method is based on the principle of total energy minimization, it can supply more realistic deformation of the catheters. In contrast to the method proposed by Alderliesten et al. our method can achieve comparable accuracy and much faster performance to make the simulator run in an interactive manner.

The rest of this paper is organized as follows.  Section 2 provides the details on the physically based deformable model and the numerical algorithm for simulating catheters.  Section 3 reports experiments and evaluation results. Finally, conclusions are drawn in Section 4.

## 2. Method

### 2.1. Total Potential Energy of a Catheter

During VIR interventions, a catheter is confined inside blood vessels and advanced along vasculature driven by the operations from the interventionalists. It is observed that a catheter would, regardless of what operations are performed on it, trend to reach an equilibrium state and finally be static if there are no continuing inputs, which can be well explained by the principle of minimum potential energy. That is the catheters would deform or displace to a position that minimizes the total potential energy. Therefore, we can solve the position and shape of the catheter by minimizing its potential energy.

We can define the total potential energy *U* of a catheter in the vascular interventional circumstance as the sum of three different components: the elastic energy *U*_*e*_ deriving from the bending of the catheter, the potential energy *U*_*p*_ generated by the interactions with the blood vessel wall, and the work *W* by external forces (e.g., the frictions and the forces from the users) acted on the catheter:

(1) $$\begin{array}{c} U=U_{e}+W+U_{p}. \end{array}$$

### 2.2. The FEM-Based Numeric Solver for Interactive Catheter Simulation

We simulate the dynamics of a catheter during VIR procedures by employing a FEM- [16] based numerical solver, where the continuous catheter can be discretized into a set of elements (segments with two end nodes in our case), and thus the degree of freedom (DoF) (positions and tangents of nodes in our case) of the catheter can be limited. We proposed a series of methods to formulate the three aforementioned energy terms in the form of quadratic polynomial functions of the tangents of the discrete catheter. To minimize the total potential energy, we calculate the partial derivative of the quadratic polynomial functions with respect to the tangents and then build a linear system. By solving this linear system, we achieve the solution with minimal potential energy.

#### 2.2.1. Formulation of Elastic Energy

First, we formulate the bending energy based on the Kirchhoff's theory of elastic rod [17, 18]. The Kirchhoff's theory is widely used in mechanics to formulate the elastic energy of deformed thin objects. In general, the elastic energy includes bending energy and twisting energy. However, in catheterization procedures, catheters have excellent torque controls, and it is usually assumed that the torsion constant of the catheters approaches infinity [12]. As a result, the twist is not taken into consideration when we formulate the elastic energy. Thus, the elastic energy *U*_e_ of the catheter can be defined as

(2) $$\begin{array}{c} U_{\text{e}}=\frac{1}{2}\overset{L}{\underset{0}{\int }}\alpha {\left(x^{\prime \prime }\left(s\right)\right)}^{2}ds, \end{array}$$

where the **x**(*s*) is the function of the centerline curve of the catheter with respect to the arc length *s*, the *L* is the total length of the catheter, and the *α* is the bending constant. To avoid the difficulty in solving the second-order derivative in the energy function, we choose the tangent of the catheter's centerline to replace the function of the centerline curve. As the function of tangent **t**(*s*) is the derivative of the **x**(*s*):

(3) $$\left(t\left(s\right)=x^{\prime }\left(s\right)\right),$$

the potential energy *U*_*e*_ can be represented as

(4) $$\begin{array}{c} U_{e}=\overset{L}{\underset{0}{\int }}\alpha {\left(t^{\prime }\left(s\right)\right)}^{2}ds. \end{array}$$

**Figure 1:**
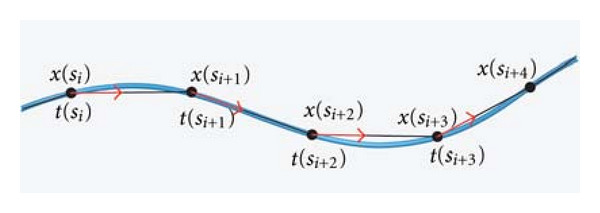
the discretized catheter with several elements.

To discretize the continuous catheter, we can apply the piecewise first-order polynomials to interpolate the function **t**(*s*). We divided the catheter into *n* elements $\left(\vec{s_{0}s_{1}},\vec{s_{1}s_{2}},\ldots ,\vec{s_{n-1}s_{n}}\right)$ as shown in Figure 1.

In the discretization, we make each element has the same length for computational convenience. The function of **t**(*s*) between nodes *s*_*i*−1_ and *s*_*i*_ can be interpolated on the segment by the first-order polynomial as follows:

(5) $$\begin{array}{c} t\left(s\right)=\frac{s-s_{i}}{s_{i-1}-s_{i}}t_{i-1}+\frac{s-s_{i-1}}{s_{i}-s_{i-1}}t_{i}. \end{array}$$

Substituting (5) into (4), we obtain the discretized energy function:

(6) $$\begin{array}{c} U_{e}=\overset{n-1}{\underset{i=0}{\sum }}\overset{s_{i+1}}{\underset{s_{i}}{\int }}\alpha {\left(t^{\prime }\left(s\right)\right)}^{2}ds. \end{array}$$

After figuring out the integral equation, the *U*_*e*_ is actually the second-order polynomial with respect to **t**_*i*_.

#### 2.2.2. Formulation of Potential Energy Caused by Interactions

Next, we formulate the potential energy *U*_*p*_ generated by the interactions with vessel walls by employing the method proposed by Alderliesten et al. [12], which is based on the Hooke's law [19]. In our simulation, blood vessels are modeled by triangular meshes [20]. During the interventional procedures, once a collision is detected, we think of it as a contact between a node of the catheter and a triangle of the vessel mesh. There should be a penetration at the contact node. We can regard the penetration as the deformation of the blood vessel wall at the contact node. Thus we can construct the formula of the *U*_*p*_ at the contact node according to the Hooke's Law: *U*_*p*_ = (1/2)*κp*_*j*_^2^, where *j* is the number of the contact nodes, *p*_*j*_ is the vertical distance from the contact node **x**_*j*_ to the contact triangle, and *κ* is the modulus of elasticity of blood vessel wall. The plane of contact triangle can be expressed as a linear equation *g*(**x**_*j*_), so the penetrating distance can be figured out as: **p**_*j*_ = |*g*(**x**_*j*_)|. Summing up all contact points, the total energy of *U*_*p*_ can be defined as

(7) $$\begin{array}{c} U_{p}=\underset{j}{\sum }\frac{1}{2}\kappa {\left|g\left(x_{j}\right)\right|}^{2}. \end{array}$$

As we can represent the **x**(*s*) with the tangent function **t**(*s*) by integrating (3):

(8) $$\begin{array}{c} x\left(s\right)=\overset{s}{\underset{0}{\int }}t\left(s\right)ds \end{array},$$

if we substitute (5) into (8) and then figure out the integration, the nodal value **x**_*i*_ can be represented by a first-order polynomial with respect to the **t**_*i*_, *i* ∈ (0,1,…, *n* − 1). Thus, the *U*_*p*_ can be transformed into a quadratic polynomial with respect to the tangent **t**_*i*_.

#### 2.2.3. Formulation of the Work by External Forces

The external forces in this application may include the frictions and the forces from the users. However, actually in clinical practice, in order to avoid the damage to the vessels of patients, the catheters are usually clothed by some biomedical materials to reduce the frictions with the blood vessel wall. The frictions are very small during the catheterization; therefore, we ignore them in our model. Hence we only take into account the forces from the users. It can be defined as

(9) $$\begin{array}{c} W=\underset{i}{\sum }f_{i}\cdot d_{i}, \end{array}$$

where **f**_*i*_ is the external force exerted on the node *i* and **d**_*i*_ is the difference between current position of node *i* and its position in the last equilibrium state. So the **d**_*i*_ can be calculated by **x**_*i*_ − **x**_*i*0_, where **x**_*i*0_ is the position of node *i* in the last equilibrium state. Also, in terms of (8), the *W* can be transformed into a quadratic polynomial with respect to the tangent **t**_*i*_.

#### 2.2.4. The Numerical Solver

In the interventional procedure, it is necessary to insert a basic sheath into the blood vessels at first. It provides safe access to the interior of the vascular network. It can be used to prevent bleeding during the procedure and restrict the direction of the catheter inserting the vessels [2]. Therefore, in our model, we regard the constant initial tangent (i.e., **t**_0_) as a boundary condition. Then, to derive the conditions of energy minimization, we calculate the partial derivative of the sum of total potential energy *U* with respect to each **t**_*i*_, *i* ∈ (1,2,…, *n* − 1) and achieve a set of linear equations which can be expressed in matrix form as

(10) $$\begin{array}{c} At=b, \end{array}$$

where **A** is a matrix by 3(*n* − 1) × 3(*n* − 1), **t** is the vector (**t**_1_^*T*^,…, **t**_*n*−1_^*T*^)^*T*^ by 3(*n* − 1) × 1, and **b** is a constant vector. During the calculation, we find that the matrix **A** can be easily transformed into an upper triangular matrix without zero element in its diagonal. As a result, it is a nonsingular matrix.

As mentioned previously, the **x**_*i*_ can be expressed as the first-order polynomial of the **t**_*i*_. This relationship can be represented in matrix form:

(11) $$\begin{array}{c} Bt=x, \end{array}$$

where **B** is a lower triangular matrix by 3(*n* − 1) × 3(*n* − 1), and there is no zero element in its diagonal, so it is a nonsingular matrix. Substituting (11) into (10), we obtain a linear system which can be expressed in matrix form:

(12) $$\begin{array}{c} AB^{-1}x=b. \end{array}$$

For every input from an interventionalist, we can reach the new equilibrium state of the catheter by solving (12).

In terms of the specific shape of the matrix *B*, the inverse matrix of **B** can be determined easily. In order to speed up our simulation, after assembling and calculating the matrix **AB**^−1^, we employ a commercial library named CUBLAS [21], which is an implementation of BLAS (basic linear algebra subprograms) on top of the NVIDIA CUDA (compute unified device architecture) driver, to solve the linear equations in our method.

#### 2.2.5. The Overall Algorithm

The overall algorithm of our solver is shown in Algorithm 1.

## 3. Experiments and Results

### 3.1. Implementation

**Figure 2:**
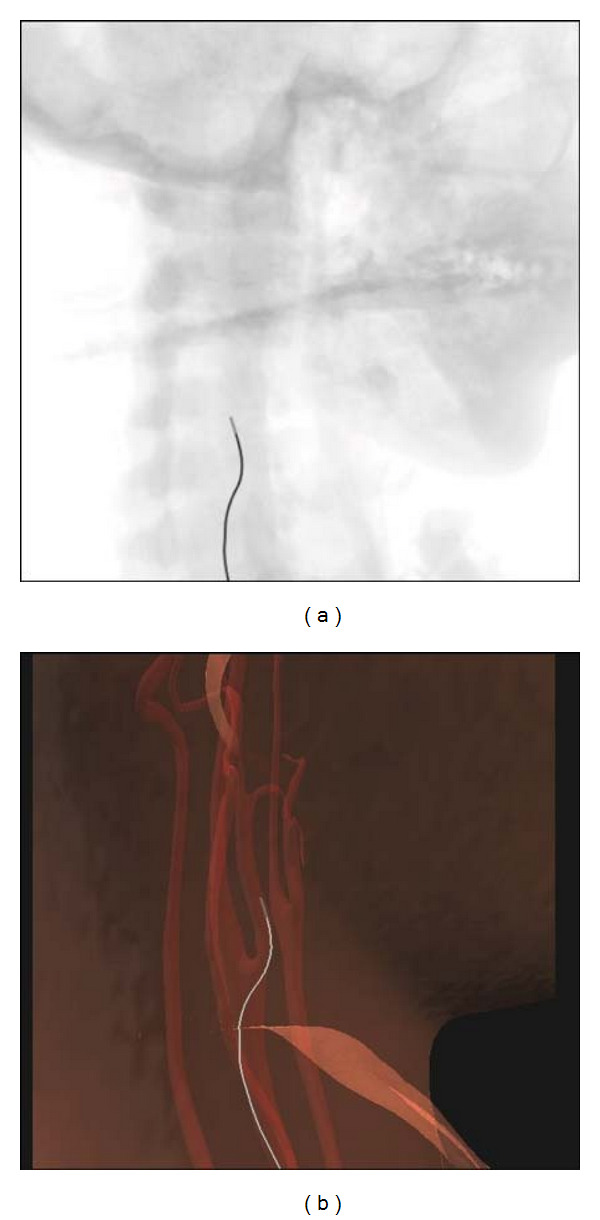
Visualization of our simulator: (a) fluoroscopic view to simulate the X-ray imaging (b) 3D anatomic view with the virtual vascular system, skin, and catheters.

We have integrated the catheters simulation into a virtual reality-based training system. It is based on a PC with a Intel Core2 6700 CPU, 4 GB memory and a NVIDIA GeForce 8800 GPU, and a hardware device made by ourself for motion sensing of the catheter. There are two views for the trainees in the system: one is the 3D navigation view, the other is the fluoroscopic view (Figure 2).

### 3.2. Experiment  1: Time Performance

In this experiment, we tested the time performance of our method for the virtual catheters with different number of nodes when they advanced in a virtual tubular blood vessel. We show the results in Table 1. As shown in the table, it takes about 36 milliseconds to complete a calculation of our algorithm for a catheter with 200 nodes. The FPS (frames per second) can be maintained about 30, which is suitable for an interactive system. Even when the number of the nodes increased to 300, the system can still reach a frame rate of 17 FPS.

### 3.3. Experiment  2: Catheters Navigation in Vascular System

**Figure 3:**
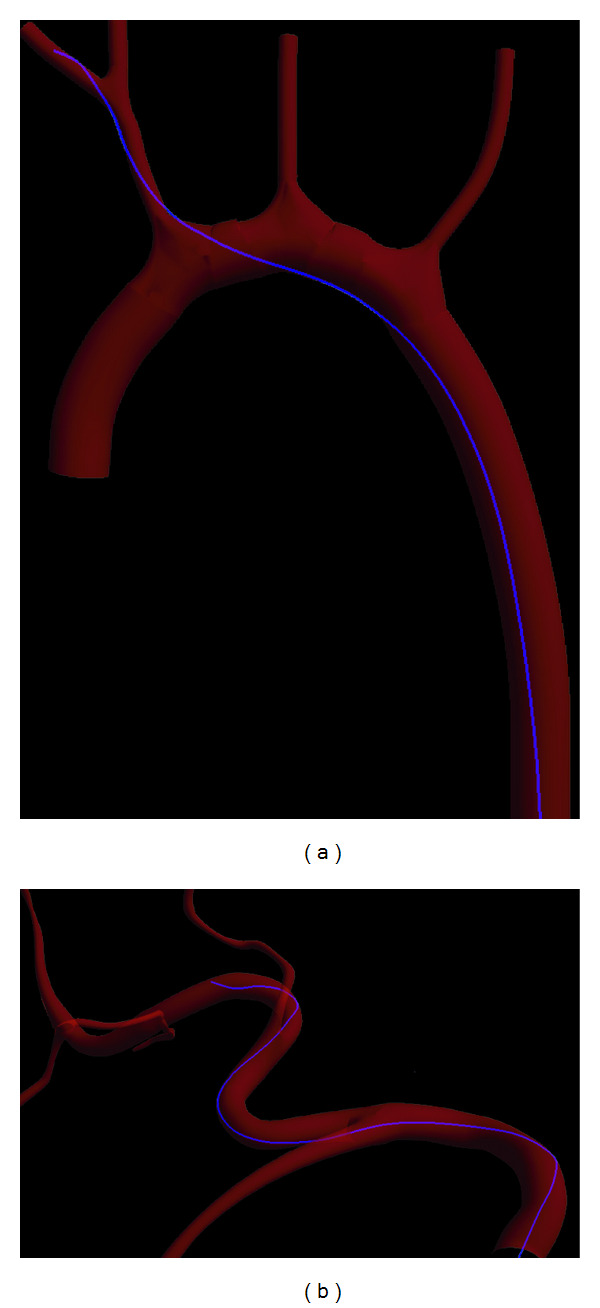
The virtual catheter navigating in different area of vascular system: (a) cardiovascular structure and (b) hepatic arterial structure.

In this set of experiment, we evaluated the capability of our method in simulating the catheters' navigation in various vascular structures. The virtual catheters modeled by our method are pushed or pulled by the interventionalist and constrained inside the vascular system. We employed the method reported in the work [22] to detect the collisions between a discretized catheter and blood vessels wall made up of triangular meshes. Under the acting force from the interventionalist and the reacting force from blood vessel walls, the catheter advances in various vascular structures. We show the snapshots of catheters' navigation in the different areas of vascular system in Figure 3.

### 3.4. Experiment  3: Behaviors of Catheters in a Vessel

We further conducted an experiment to evaluate the simulated behaviors of a catheter when moving within a blood vessel. A transparent plastic tube was used in our experiment to act like a tubular blood vessel. In this experiment, a real catheter was inserted into the plastic tube. Here, we mainly emulated a common operative situation, where the catheter would be distorted during its moving forward when its soft tip was looped back inside the blood vessel wall.

**Figure 4:**
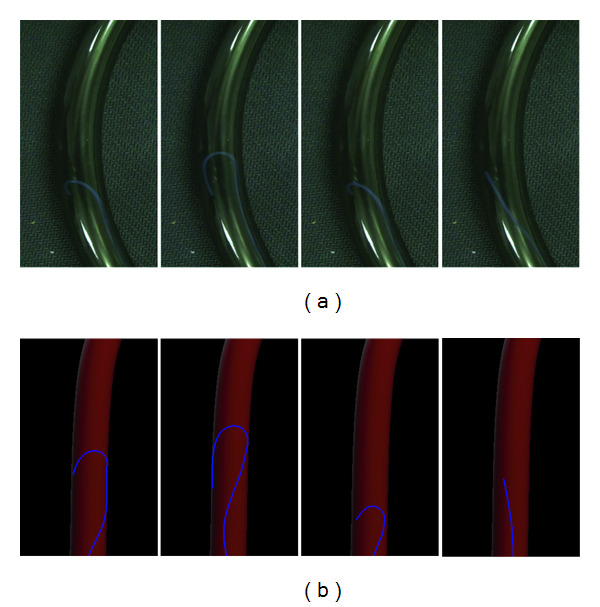
Advancement of a catheter within a tube: (a) real situation and (b) simulated results.

Figure 4 shows the results of the experiment comparing the real situation to the simulated one. From these four consecutive pictures, we can find that the distortion of the floppy region of the catheter becomes larger along its advancement. It was due to the fact that when the tip of the catheter collides with the tube, the tip was stopped from advancing so that a loop was formed. This is a very common situation which occurred in real operations. We can observe that our method can mimic this phenomenon well.

### 3.5. Experiment  4: Comparison of the Deformation between the Simulated Catheter and the Real One

**Figure 5:**
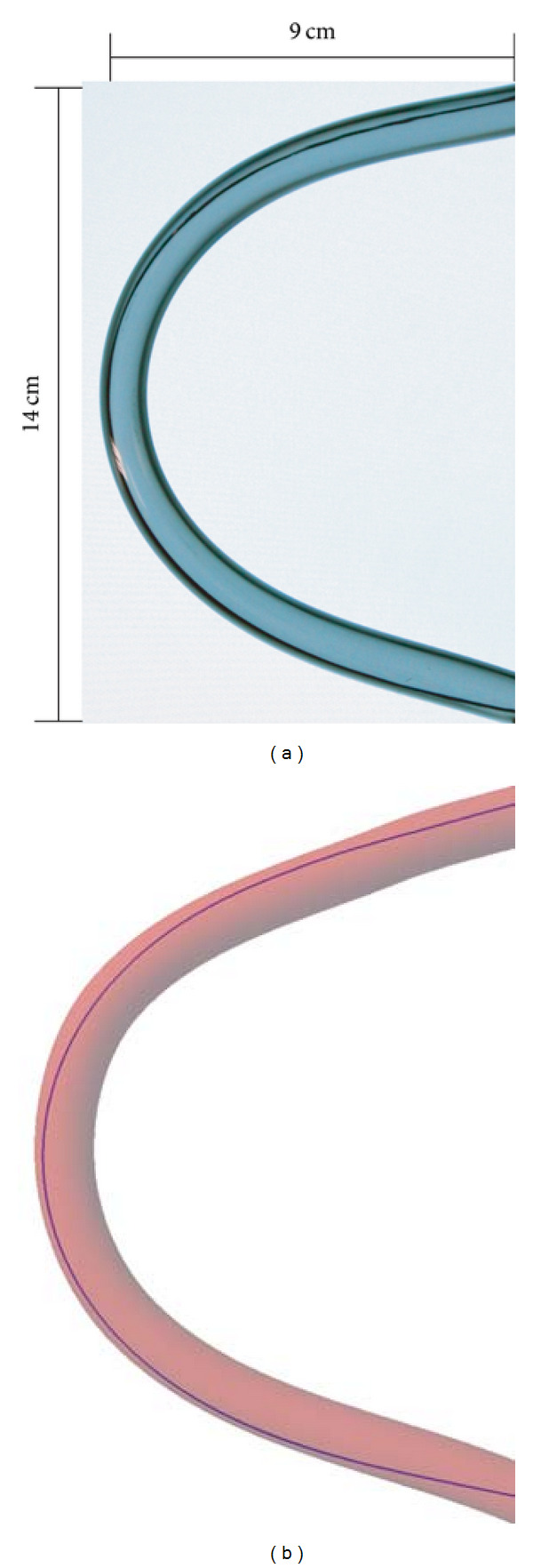
The comparison of the deformation between the simulated catheter and the real one.

Finally, we conducted a set of experiments to compare the simulated catheter advanced in the curved virtual vessel and the real one in the plastic tubular phantom to validate the realism of the deformation of the catheter in our method. The experiment for real catheter was performed to insert a real catheter into a curved plastic tube and advance it to a desired position as shown in Figure 5(a). The size of the curved plastic tube is also labeled in the figure. The shape and the position of the real catheter were acquired as the ground truth. In the virtual environment, we create a 3D model as the virtual blood vessel according to the size and the shape of the real plastic tube, and then the virtual catheter simulated by our method was inserted and advanced to the same position as shown in Figure 5(b). We acquire the position of the virtual catheter and compare them with the ground truth.

We used the root-mean-square (RMS) error to measure the difference of deformation between the real catheter and the simulated one. The RMS is computed from the distances between the nodal positions in the simulated catheter and a set of reference nodes in the ground truth. We acquired those reference points by resampling the catheter in the ground truth with the segment length used for each specific experiment. For *n* nodes, the formula of RMS is $\text{RMS}=\sqrt{(1/n)\sum_{i=0}^{n-1}{\left({\mathrm{dist}}_{i}\right)}^{2}}$, where dist⁡_*i*_ = ||*x*_*i*_^*s*^ − *x*_*i*_^*r*^||, *x*_*i*_^*s*^ is the simulated nodal position and *x*_*i*_^*r*^ is the corresponding reference nodes. In addition, we also list the maximum displacement among all of the dist⁡_*i*_ to measure the difference of deformation. In Table 2, besides RMS and the maximum displacement, we also list the total runtime in seconds of the whole procedure of the experiments.

The results can be compared with the experiment data in the work of Alderliesten et al. [13]. It can be observed that the error of our method is slightly bigger than their results, but the time performance is much better than their method. For example, when the segment length is 1 mm and the ratio of the stepsize to the segment length is 1/10, the runtime of our results is 41.4 seconds, while the runtime in the work of Alderliesten et al. [13] is 2117.3 seconds. According to our experimental results, We can find that the errors are becoming smaller with the reduction of the segment length, however the runtime is increasing correspondingly. Therefore, there is a trade-off between the accuracy and efficiency. We should choose the segment length as small as possible, at the same time make the simulator run in the real-time interactive manner.

## 4. Conclusion and Discussion

The VR-based surgical simulator is widely applied to teach and train the medical students. It is indispensable to make the simulator interact with the trainees with real-time response. Beyond that, the simulator should provide an as realistic virtual environment as possible in which trainees can fully immerse themselves as if they were in real operating scenarios. In the catheterization, the simulation of behaviors of catheters is a very important and relatively complex component of a VR simulator. In this paper, we are dedicated to building the physically deformable model for the simulation of the catheters and simulating the interaction between the catheters and the blood vessel wall. In our method, we regard the motion of the catheter as the transition from an equilibrium state to another; therefore, we formulate the potential energy function relevant to the elastic property of the catheter, the deformation of the blood vessel wall, and the work by external forces. We resort to the concept of the FEM to construct and solve a linear system to achieve the new equilibrium state of the catheter with responses to each input from the interventionalist. Our method is integrated into a simulator for the training of VIR surgeries, and the behavior of the catheter simulated by our method can make the training process realistic.

However, in our proposed method we do not take into consideration the twisting problem of the catheter which is useful for the simulation of some other VIR procedure, such as embolization. Therefore, we will adopt the concept of the frames to represent the twisting state of the catheter and adapt our potential energy function for involving the twisting energy in the future. Furthermore, by improving our deformable model, we will extend our method to simulate other devices such as coils as well as the embolization process which is performed to cure the arterial aneurysm by deploying the coils in the aneurysm.

In the future, another work of us is to evaluate our virtual system by means of empirical study approach. In details, we will design a set of specific training subject in our virtual system based on the real catheterization procedure. And the virtual angiography procedure will also be integrated into our system, so that the trainees can practice the catheterization procedure under the guide of the simulated 2D X-ray imaging. We will invite the medical students and some specialists of interventionalists to participate our experiments. Then, we will let them complete a series of experiments and analyze the results to estimate the validity for training of our virtual system.

## Figures and Tables

**Algorithm 1:**
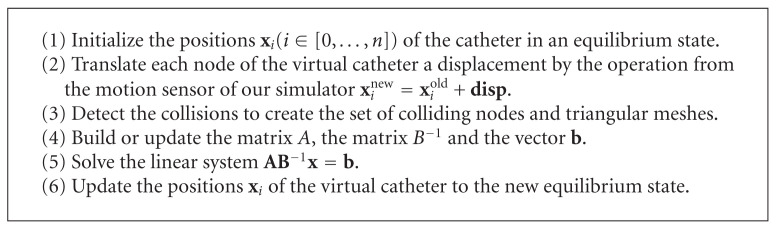
The overall algorithm of the proposed numerical solver.

**Table 1:** Timing performance of our method.

Number of node	Average execution time (ms)	Frames per second
50	9.2	109
100	15.4.	65
150	24.8	40
200	36.7	27
300	58.4	17

**Table 2:** The comparison of the deformation between the simulated catheter and the real one: for different combinations of segment length *l* and stepsize of each input (pulling or pushing) *h*, the RMS error (mm) (left value), the maximum displacement error (mm) (middle value), and the total runtime of the whole procedure of the experiments in seconds (right value) are listed.

*h*/*l*	*l*								
	1 mm			2 mm			3 mm		
1/40	1.38	2.32	180.7	1.54	2.42	49.7	1.76	2.74	29.5
1/20	1.16	2.12	84.5	1.47	2.24	23.2	1.66	2.38	15.4
1/10	1.12	2.16	41.4	1.35	2.36	11.5	1.86	2.45	6.9
1/5	0.96	1.52	20.5	1.26	1.88	5.9	1.44	2.56	3.9
1/3	1.05	1.76	11.6	1.24	2.13	3.5	1.62	2.22	2.1
